# Evolutionary engineering and molecular characterization of an antimycin A-resistant *Saccharomyces cerevisiae* strain: the key role of pleiotropic drug resistance (*PDR1*)

**DOI:** 10.1093/femsyr/foaf062

**Published:** 2025-10-17

**Authors:** Alican Topaloğlu, Can Holyavkin, Ömer Esen, Ogün Morkoç, Karl Persson, Cecilia Geijer, Zeynep Petek Çakar

**Affiliations:** Faculty of Science and Letters, Department of Molecular Biology and Genetics, Istanbul Technical University, Maslak, Istanbul 34469, Türkiye; Dr. Orhan Ocalgiray Molecular Biology, Biotechnology and Genetics Research Center (ITU – MOBGAM), Istanbul Technical University, Maslak, Istanbul 34469, Türkiye; Faculty of Science and Letters, Department of Molecular Biology and Genetics, Istanbul Technical University, Maslak, Istanbul 34469, Türkiye; Dr. Orhan Ocalgiray Molecular Biology, Biotechnology and Genetics Research Center (ITU – MOBGAM), Istanbul Technical University, Maslak, Istanbul 34469, Türkiye; Faculty of Science and Letters, Department of Molecular Biology and Genetics, Istanbul Technical University, Maslak, Istanbul 34469, Türkiye; Dr. Orhan Ocalgiray Molecular Biology, Biotechnology and Genetics Research Center (ITU – MOBGAM), Istanbul Technical University, Maslak, Istanbul 34469, Türkiye; Dr. Orhan Ocalgiray Molecular Biology, Biotechnology and Genetics Research Center (ITU – MOBGAM), Istanbul Technical University, Maslak, Istanbul 34469, Türkiye; Faculty of Science, Department of Molecular Biotechnology, Turkish-German University, Beykoz, Istanbul 34820, Türkiye; Division of Industrial Biotechnology, Chalmers University of Technology, 41296 Gothenburg, Sweden; Division of Industrial Biotechnology, Chalmers University of Technology, 41296 Gothenburg, Sweden; Faculty of Science and Letters, Department of Molecular Biology and Genetics, Istanbul Technical University, Maslak, Istanbul 34469, Türkiye; Dr. Orhan Ocalgiray Molecular Biology, Biotechnology and Genetics Research Center (ITU – MOBGAM), Istanbul Technical University, Maslak, Istanbul 34469, Türkiye

**Keywords:** adaptive laboratory evolution, antifungal drug resistance, antimycin A, evolutionary engineering, CRISPR/Cas9, pleiotropic drug resistance (*PDR1*)

## Abstract

Antimycin A, an antifungal agent that inhibits mitochondrial respiration, provides a useful model for studying resistance mechanisms. Antifungal resistance is an escalating clinical concern with limited treatment options available. To understand the molecular mechanisms of antimycin A resistance, a genetically stable, antimycin A-resistant *Saccharomyces cerevisiae* strain was successfully developed for the first time through an evolutionary engineering strategy, based on long-term systematic application of gradually increasing antimycin A stress in repetitive batch cultures without prior chemical mutagenesis. Comparative whole genome resequencing analysis of the evolved strain *ant905-9* revealed two missense mutations in *PDR1* and *PRP8* genes involved in pleiotropic drug resistance and RNA splicing, respectively. Using CRISPR/Cas9 genome editing tools, the identified mutations were introduced individually and together into the reference strain, and it was confirmed that the Pdr1p.M732R mutation alone confers antimycin A-resistance in *S. cerevisiae*. Comparative transcriptomic analysis of the reverse-engineered Pdr1p.M732R strain showed alterations in PDR (pleiotropic drug resistance), transmembrane transport, vesicular trafficking, and autophagy pathways. Our results highlight the potential key role of *PDR1* in antifungal drug resistance. This study provides new insights into mitochondrial drug resistance and the adaptive potential of yeast under respiratory stress.

## Teaser

Evolutionary engineering of an antimycin A-resistant *Saccharomyces cerevisiae* strain, comparative omic analyses, and reverse engineering using CRISPR/Cas9 revealed the importance of PDR1 gene in antifungal drug resistance.

## Introduction

Antimycin A is an antifungal drug isolated from *Streptomyces* species (Dunshee et al. 1949). It consists of 3-formyl aminosalicylic acid linked to a dilactone ring via amide bonds bridge. Structurally similar antimycin A complexes differ in the length and branching of the alkyl and acyl groups on the dilactone ring (van Tamelen et al. 1961). As a potent inhibitor of mitochondrial respiration, antimycin A exerts significant effects across a wide spectrum of organisms, ranging from the yeast *Saccharomyces cerevisiae* to higher eukaryotes including fish, mice, and humans, even at nanomolar concentrations (Ott 1994, Saari 2023). Commercially, antimycin A has been used in aquaculture under the trade name “Fintrol^®^,” to control unwanted fish species (Hubert and Schmidt 2001). More recently, there has been a growing interest in the use of antimycin A as a natural and strong biopesticide in agricultural applications: a marine antimycin A was shown to suppress wheat blast disease (Paul et al. 2022), and an antimycin A isolated from *Streptomyces* species found in the rhizosphere soil of an ancient banyan tree was shown to be a potent inhibitor of the plant pathogenic fungus *Rhizoctonia solani* (Zhu et al. 2024).

Antimycin A inhibits respiration by binding to the mitochondrial complex III and blocks electron transfer between cytochromes b and c, which leads to the generation of oxygen free radicals due to the interrupted electron flow (Rieske 1980, Balaban et al. 2005). As the mitochondrial complex III affects 80% of reactive oxygen species (ROS) generation, antimycin A can affect oxidative stress, aging and autophagy-related pathways (Grant et al. 1997, Ma et al. 2011). Its potential as a therapeutic agent against some cancer types stems from a ROS-induced apoptotic behavior (Park and You 2016).

As a facultative anaerobe, the yeast *S. cerevisiae* is a suitable eukaryotic model organism to study mitochondrial stress and mitochondrial dysfunction, which is involved in the pathophysiology of diverse diseases and the aging process (Zong et al. 2024). *Saccharomyces cerevisiae* also has survival mechanisms against mtDNA mutations (Lasserre et al. 2015). Despite the diverse applications of antimycin A as a strong antifungal drug and a potential therapeutic agent as a strong inhibitor of mitochondrial respiration, there is limited information on yeast stress response and resistance mechanisms against antimycin A. A previous study focused on the transcriptomic response of *S. cerevisiae* cells to short-term antimycin A treatment, which revealed that the energetically costly pathways, such as protein synthesis, DNA and RNA processing, and ribosomal biogenesis were downregulated, and glycolysis and reserve energy metabolism pathways (trehalose/glycogen synthesis) were upregulated, emphasizing the role of the environmental stress response gene networks in response to respiratory inhibition by antimycin A (Lai et al. 2008). To the best of our knowledge, however, there are no reports in the literature on the molecular mechanisms of antimycin A resistance in *S. cerevisiae* strains that are resistant to antimycin A.

Evolutionary engineering or adaptive laboratory evolution is a systematic and powerful strategy based on random mutation and selection to improve genetically complex microbial traits, including stress resistance (Sauer 2001, Çakar et al. 2012, Dragosits and Mattanovich 2013). It has been successfully used to improve biofuels and chemicals production (Mans et al. 2018, Topaloğlu et al. 2023), and to develop robust *S. cerevisiae* strains that are resistant against industrially important stress factors, such as oxidative stress (Kocaefe-Özşen et al. 2022), starvation stress (Arslan et al. 2018), ethanol (Turanlı-Yıldız et al. 2017, Mavrommati et al. 2023), coniferyl aldehyde (Hacısalihoğlu et al. 2019), and 2-phenyl ethanol (Holyavkin et al. 2023). Owing to the rapid developments in high-throughput “omics” technologies, the evolved microbial strains can be characterized in detail and the complex genetic basis of the desired traits can be identified using reverse engineering strategies, which include genome editing tools such as CRISPR/Cas9 technology (Mans et al. 2018, Topaloğlu et al. 2023).

The aim of this study was to gain insight into the molecular mechanisms of antimycin A resistance in *S. cerevisiae*. For this purpose, a haploid laboratory reference strain of *S. cerevisiae* was subjected to gradually increased antimycin A stress in successive batch cultures under respiratory growth conditions using an evolutionary engineering strategy, and a genetically stable, antimycin A-resistant evolved strain was obtained. The evolved strain was physiologically characterized and comparative transcriptomic and genomic analyses were performed. To clarify the roles of the identified genomic changes in the evolved strain, those single nucleotide variations (SNVs) were introduced individually and in combination to the background strain using CRISPR/Cas9 genome editing technology, and the resulting strains were tested for antimycin A resistance. Using this comprehensive reverse engineering approach and omic analyses, we have shown that the *PDR1* gene and the pleiotropic drug resistance (PDR) pathway are responsible for antimycin A-resistance in *S. cerevisiae*.

## Materials and methods

### Strains and growth conditions

The haploid *S. cerevisiae* strain CEN.PK 113–7D (*MATa, MAL2–8^c^, SUC2*), was kindly provided by Professor Dr Jean Marie Francois and Dr Laurent Benbadis (University of Toulouse, France), and used as the reference yeast strain in this study (Entian and Kötter 2007). For respiratory growth conditions, yeast cells were cultivated in shake flasks, using YNBE medium [0.67% (w/v) yeast nitrogen base without amino acids, 1% (w/v) ethanol, pH 6.0] at 30°C and 150 rpm. For fermentative growth, yeast minimal medium [YMM; 0.67% (w/v) yeast nitrogen base without amino acids, 2% (w/v) d-glucose, pH 6.0] was used, and yeast cells were grown in culture tubes at 30°C and 150 rpm in an orbital shaker. In reverse engineering steps, YP (yeast extract-peptone) medium [1% (w/v) yeast extract and 2% (w/v) peptone] with 2% (w/v) d-glucose or galactose as the carbon source was used, supplemented with antibiotics (200 µg/ml hygromycin and 200 µg/ml geneticin), when necessary. *Escherichia coli* strain DH5α cultures were grown in Luria–Bertani medium [0.5% (w/v) tryptone, 0.5% (w/v) NaCl, and 0.25% (w/v) yeast extract] (Bertani 1951), supplemented with antibiotics (100 µg/ml neomycin and 100 µg/ml ampicillin). For culturing on solid media, agar was added to a final concentration of 2% (w/v). Cell growth in liquid cultures was monitored by optical density (OD) measurements at 600 nm wavelength, using a spectrophotometer (Shimadzu UV-1700, Japan). Culture stocks were stored at −80°C in 30% (v/v) glycerol.

### Evolutionary engineering procedure

Evolutionary selection was applied in serial batch cultures under respiratory growth conditions, at gradually increasing levels of antimycin A stress. Initially, the overnight preculture of the reference strain CEN.PK 113–7D was inoculated into 20 ml of YNBE medium supplemented with 0.1 nM antimycin A in a 100 ml shake flask to an initial OD_600_ of 0.2 (∼2.8 × 10^6^ cells/ml) and grown for 24 h at 30°C and 150 rpm, in parallel with the same culture grown in YNBE medium without antimycin A, to serve as a control. Daily passages were prepared similarly and continued with increasing antimycin A concentration by 0.1–0.2 nM at each successive passage, up to 6.6 nM antimycin A at the final (52nd) passage or population of selection. Survival rates of the cultures were calculated by dividing the OD_600_ of the culture under antimycin A-stress by the OD_600_ of the culture under control (nonstress) conditions, as described previously (Arslan et al. 2018) and culture stocks were stored at −80°C in 30% (v/v) glycerol for each passage. The last population of selection was spread on 7.5 nM antimycin A-containing YNBE plates and resistant colonies were randomly picked after 72 h of incubation at 30°C.

### Stress resistance estimation

Resistance of the mutant strains and the reference strain against antimycin A and other stressors was estimated by spot assay, as described previously (Sürmeli et al. 2019). Briefly, precultures of the evolved strain and the reference strain were inoculated in 10 ml YMM in 50 ml culture tubes starting from 0.2 OD_600_ (∼2.8 × 10^6^ cells/ml) and grown until the mid-exponential phase of growth (∼10^7^ cells/ml) at 30°C and 150 rpm. Cells were then harvested by centrifugation (10 000 × *g* for 5 min) and resuspended in dH_2_O to have a cell density of 4 OD_600_/ml. The cell suspensions were serially diluted by 10-fold up to 10^−5^ dilution and 5 µl samples were spotted onto YMM plates that contained the following stressors:15 mM caffeine (Merck, Darmstadt, Germany), 150 µg/ml propolis (from Kartal, Istanbul, Türkiye), 1 mM coniferyl aldehyde (Merck), 200 ng/ml cycloheximide (Merck), 4 mM vanillin (Merck), 8% (w/v) ethanol (J.T Baker, Deventer, Netherlands), 10% (w/v) methanol (J.T Baker), 2 g/l 2-phenylethanol (Merck), 2.5 mM H_2_O_2_ (Merck), 50 mM NH_4_Fe(SO_4_)_2_ (Merck), 2.5 mM CrCl_3_ (Acros Organics, Fair Lawn, NJ, USA), 3 mM CoCl_2_ (Fluka, Charlotte, NC, USA), and 10 mM AlCl_3_ (Merck). In spot assays for antimycin A resistance estimation, YNBE medium and YNBE solid plates were used that contained 2.5 and 15 nM antimycin A. The plates were incubated at 30°C for 72 h. All experiments were performed in three biological replicates.

### Growth physiological analyses

The growth physiology of the evolved strain in comparison with the reference strain was analysed in batch cultures under both fermentative (YMM) and respiratory growth conditions (YNBE with or without 1 nM antimycin A supplementation). The precultures of the evolved and reference strains were inoculated into 200 ml medium in 1 l shake flasks, starting from 0.2 OD_600_ (∼2.8 × 10^6^ cells/ml) and cultivated in an orbital shaker at 150 rpm and 30°C for 72 h. At regular time intervals, samples for absorbance (OD_600_), cell dry weight, extracellular metabolite analysis, and storage carbohydrate analysis were taken. Residual glucose, glycerol, acetate, and ethanol concentrations were determined by using a Shimadzu Series 10A HPLC system (Shimadzu Co., Kyoto, Japan), equipped with a RID-10A refractive-index detector and an Aminex HPX-87H ion exclusion column (300 mm × 7.8 mm, Bio-Rad Laboratories, CA, USA) working at 65°C and 5 mM H_2_SO_4_ as a mobile phase with a flow rate of 0.6 ml/min. The intracellular trehalose and glycogen levels were determined according to Divate et al. (2016) and Parrou and François (1997), with minor modifications. Basically, 1 ml of samples were taken, centrifuged (10 000 × *g* for 3 min) and washed with dH_2_O. For trehalose analysis, samples were resuspended in 1 ml dH_2_O and incubated at 95°C for 1 h prior to high-performance liquid chromatography (HPLC) analysis. For glycogen analysis, samples were resuspended in 0.25 ml 0.25 M Na_2_CO_3_ and incubated at 95°C for 4 h. The pH was adjusted to 5.2 by adding 0.15 ml 1 M acetic acid and 0.6 ml 0.2 M sodium acetate (pH 5.2). The samples were incubated overnight, shaken at 57°C and 300 rpm, with 1.2 U/ml amyloglucosidase (11202332001; Roche Diagnostics) to release glucose prior to HPLC analysis. All physiological experiments were performed in three biological replicates.

### Cell wall integrity analysis

The cell wall integrity of the evolved strain and the reference strain was assessed by lyticase sensitivity assay adapted from Kuranda et al. (2006), as described previously (Sürmeli et al. 2019). The precultures of the evolved and the reference strains were grown in 250 ml shake flasks containing 50 ml YNBE medium with or without 1 nM antimycin A at 30°C and 150 rpm, starting from an initial OD_600_ of 0.2 (∼2.8 × 10^6^ cells/ml) until the stationary phase of growth (∼6.0 × 10^7^ cells/ml). The stationary phase cells were then collected by centrifugation (5500 × *g* for 10 min) and resuspended in 10 ml of 10 mM Tris/HCl buffer (pH 7.4) supplemented with 40 mM β-mercaptoethanol, to an OD_600_ of 0.9 per ml. After incubation at 25°C for 30 min, 2 U/ml lyticase was added and cell lysis was monitored by measuring the absorbance (OD_600_) every 20 min. The measured absorbance values were divided by the initial absorbance value and the results were multiplied by 100 to calculate the % lyticase resistance.

### Whole genome resequencing analysis

For the whole-genome resequencing analysis of the reference strain and the evolved strain, cells were grown in liquid yeast extract-peptone-dextrose (YPD) medium (2% (w/v) dextrose, 2% (w/v) peptone, and 1% (w/v) yeast extract, pH 6.0) until the stationary phase of growth (∼6.0 × 10^7^ cells/ml), cells were harvested (5500 × *g* for 10 min), and total DNA was isolated using MasterPureTM DNA Purification Kit (Epicenter, San Diego, USA), according to the manufacturer’s instructions. The quality and quantity of isolated DNA samples were determined by using NanoDrop 2000 UV–Vis spectrophotometer (Thermo Fisher Scientific, Waltham, USA). Prior to sequencing, genomic libraries were prepared by using Ion Xpress Plus Fragment Library Kit (Thermo Fisher Scientific) and Ion 540^TM^ Chip Kit (Thermo Fisher Scientific). Sequencing was performed on the Ion S5 Next-Generation Sequencing Platform (Thermo Fisher Scientific) coupled with the automated library prep platform Ion Chef (Thermo Fisher Scientific). The quality check was done by FastQC (v.0.11.5) software (Babraham Bioinformatics) and adapter sequences/low-quality reads were removed using Trimmomatic (v.0.32) software (Bolger et al. 2014). For the alignment of read sequences to *S. cerevisiae* CEN.PK113–7D reference genome (GCA 000269885.1), Burrows–Wheeler aligner MEM v.0.7.1 (Li and Durbin 2009) was used and mutations were called using Genome Analysis Toolkit (v.3.8.0) (DePristo et al. 2011). Nucleotide changes were inspected on Genome-Browse (v.2.1.2) (GoldenHelix). High-quality point mutations were filtered using in-house R scripts (R Core Team 2020) and unique mutations found in the evolved strain were annotated by using Variant Effect Predictor (v.90) using the latest gene build *S. cerevisiae* CEN.PK113-7D, ASM26988v1. Whole-genome resequencing data have been deposited in the NCBI Sequence Read Archive (SRA) database under BioProject PRJNA1166665.

### Reverse engineering using CRISPR/Cas9

Targeted genome modifications were adapted from Mans et al. (2015), with minor changes. The yeast repair machinery was used to integrate Pdr1p.M732R and Prp8p.V2218 L point mutations to the *S. cerevisiae* CEN.PK 113–7D background (reference) strain. For both point mutations, gRNA sequences targeting Cas9 to cutting sites, modified dsDNA repair fragments and primers for validation of point mutations and gRNA integrations (Supplementary file 1) were designed using the free online tools Benchling (Benchling 2021; https://www.benchling.com/) and CHOPCHOP softwares (Labun et al. 2019), and confirmed manually on SnapGene® software (SnapGene® software 2025; from Dotmatics). gRNA spacer sequences were integrated into the pMEL13 plasmid (Addgene # 107919) by substitution polymerase chain reaction (PCR), using Phusion High Fidelity Taq Polymerase (Thermo Fisher Scientific), cleaned up by using GeneJET PCR Purification Kit (Thermo Fisher Scientific) and amplified in *E. coli*. Amplified plasmids were isolated using GeneJET Plasmid MiniPrep Kit (Thermo Fisher Scientific) and correct gRNA integrations were verified by colony PCR using diagnostic primers (Supplementary file 1). *Saccharomyces cerevisiae* CEN.PK 113–7D reference strain was transformed according to Gietz and Woods (2002), with minor modifications. The background strain was first transformed with aCas9 plasmid assembled in-house using the MoClo Modular Cloning System Plasmid Kit (Lee et al. 2015), where the Cas9 gene is under the control of the galactose-inducible promoter GAL1p and carrying the G418 marker. Transformants carrying Cas9 plasmid were used for the second round of transformation with constructed pMEL13 plasmids carrying gRNAs for Pdr1p.M732R and Prp8p.V2218 L cutting sites. Finally, the transformants were subjected to a third round of transformation by modified dsDNA repair fragments. Transformed cells were plated onto YP-galactose agar medium supplemented with necessary markers for pMEL13 (200 µg/ml hygromycin B) and Cas9 plasmids (200 µg/ml geneticin) to induce Cas9 enzyme. The transformants grown on the YP-galactose plates were restreaked on YPD agar plates and verified by colony PCR to check the Pdr1p.M732R and Prp8p.V2218 L point mutations (Lõoke et al. 2011). In the positive mutants, the PCR products, an ~300 bp region (both up- and downstream) surrounding the point mutation site were sequenced to confirm the successful insertion of the point mutations. The double mutation-carrying strain was also constructed by the same methodology, using the Prp8.V2218 L transformant as the background strain.

### Whole genome transcriptomic analysis

For the comparative whole-genome transcriptomic analysis of the reverse engineered, Pdr1p.M732R mutant strain and the reference strain, Illumina NextSeq 550 Next-Generation Sequencing Platform was used. Transcriptomic material was collected in three biological replicates from both the evolved and the reference strain, using RNeasy Mini Kit (Qiagen, German Town, USA), after growing the cultures up to 1.0 OD_600_ (∼10^7^ cells/ml) in 100 ml YNBE medium in 500 ml shake flasks. The isolated RNA samples’ quality and quantity were measured using Nanodrop 2000 UV–Vis spectrophotometer (Thermo Fisher Scientific). The RNA integrity numbers (RIN) were determined using Agilent Bioanalyzer 2100 and the Agilent RNA 6000 Nano Assay Kit (Agilent Technologies, Santa Clara, USA). Samples with RIN values higher than 7.0 were included in the transcriptomic analyses. The libraries were created using the Illumina Stranded mRNA Prep Kit, to enrich the mRNAs containing poly-A sequences from the isolated RNA samples to make them ready for sequencing. Illumina NextSeq 550 Next-Generation Sequencing Platform was used for the sequencing experiments. Quality control of the obtained raw data was performed using FastQC software (Babraham Bioinformatics) and low-quality reads were trimmed using the Trimmomatic (Bolger et al. 2014) software. Postclipping reads were aligned with the Tophat software (Trapnell et al. 2009). After alignment, the total number of reads on each transcript were calculated and then normalized to the total number of reads. R-scripts were coded within the scope of the project and used in comparison studies and data visualization applications between the reference and the evolved strains. The Gene Ontology (GO) enrichment and KEGG pathway analysis were performed using DAVID Bioinformatics Resources (v6.8) (Huang et al. 2009a, b) for the significantly altered transcripts (*P* ≤ .05 and fold change ≥ 2). Raw data were deposited in the NCBI SRA database under the accession number SRP535472.

### Statistical analysis

All experiments in this study, except whole genome resequencing were performed in at least three biological replicates. Data analyses were conducted using the “stats” package (v.4.3.1) on R software (R Core Team 2020). Statistical significances (*P* < .05) were determined by Student’s *t*-test (two-tailed, unpaired).

## Results

### Isolation of antimycin A-resistant strains by evolutionary engineering

**Figure 1.**
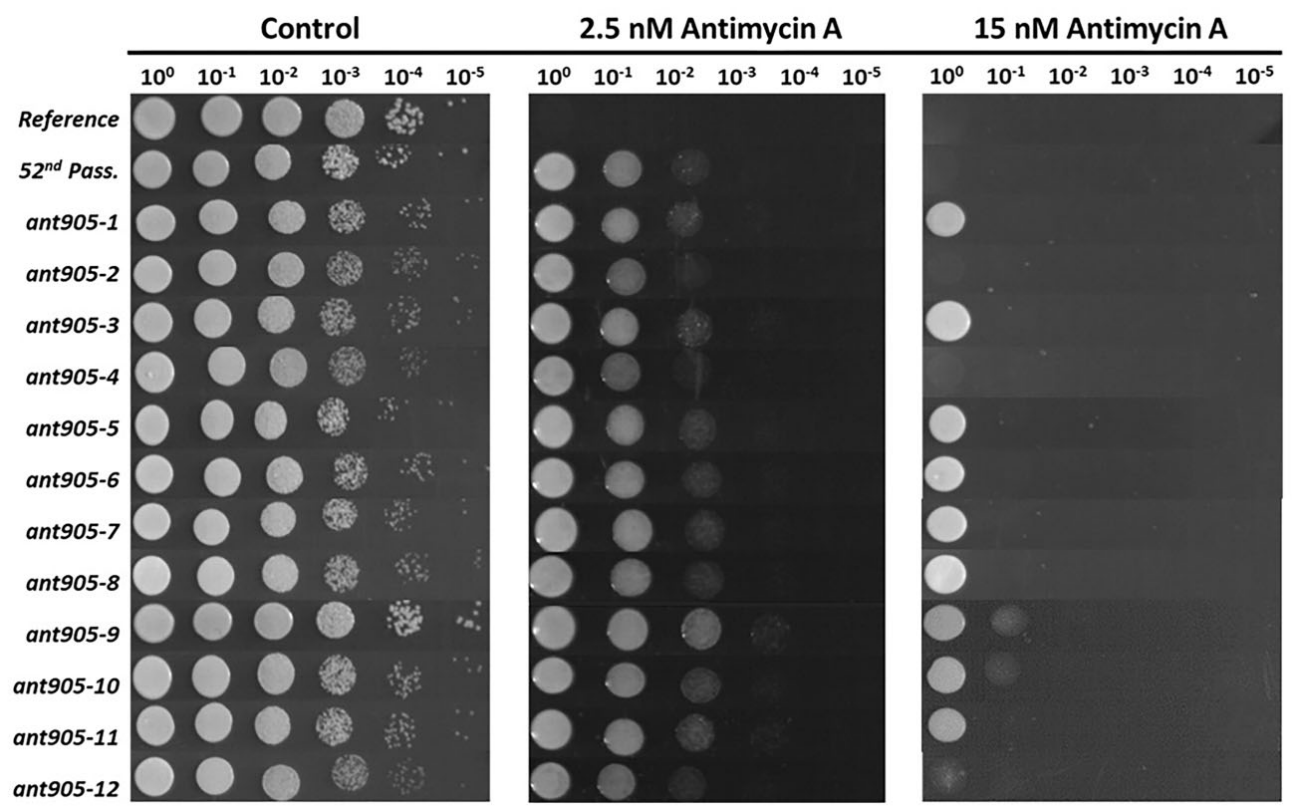
Antimycin A-resistance of selected evolved strains (*ant905-1* to *ant905-12*), the reference strain and the final passage (final population) of selection (*52nd Pass*.) assessed by using the spot assay in YNBE agar supplemented with 2.5 and 15 nM antimycin A. Images were taken at the 72nd hour of incubation at 30°C.

*Saccharomyces cerevisiae* CEN.PK 113–7D haploid laboratory reference strain was evolved using an evolutionary engineering strategy, to generate a genetically stable and highly antimycin A-resistant strain. Prior to selection experiments, it was found that 1 nM antimycin A in liquid YNBE media resulted in 50% decrease in final OD_600_ values of the reference strain after 24 h of incubation at 30°C and 150 rpm. To start with a mild stress level, 0.1 nM antimycin A was chosen as the initial stress level of the evolutionary selection experiments. The selection procedure was performed as serial batch cultivations in respirative YNBE medium with gradually increasing concentrations of antimycin A from 0.1 nM to 6.6 nM through 52 successive passages or populations. From the final (52nd) population, 12 individual colonies were isolated randomly on 7.5 nM antimycin A-containing YNBE agar plates. Their antimycin A-resistance was further assessed by spot assays, using YNBE agar plates supplemented with 2.5 and 15 nM antimycin A concentrations at which the reference strain could not grow at all (Fig. 1). Among the resistant candidates, the *ant905-9* strain with a higher antimycin A-resistance than the other mutant individuals was chosen for further analysis. This strain and the reference strain were cultivated in liquid YNBE at varying antimycin A concentrations. While the reference strain could not survive at antimycin A levels above 1 nM, the *ant905-9* strain had a survival rate of about 40% at 25 nM antimycin A stress condition, upon 24 h of incubation at 30°C and 150 rpm. (Fig. S1). In addition, it was verified that *ant905-9* was genetically stable, as it retained its antimycin A resistance upon 10 successive passages in a nonselective medium followed by stress resistance estimation for growth on YNBE agar plates supplemented with 7.5 nM antimycin A (Fig. S2).

### Cross-resistance of the evolved strain against other stressors

**Figure 2.**
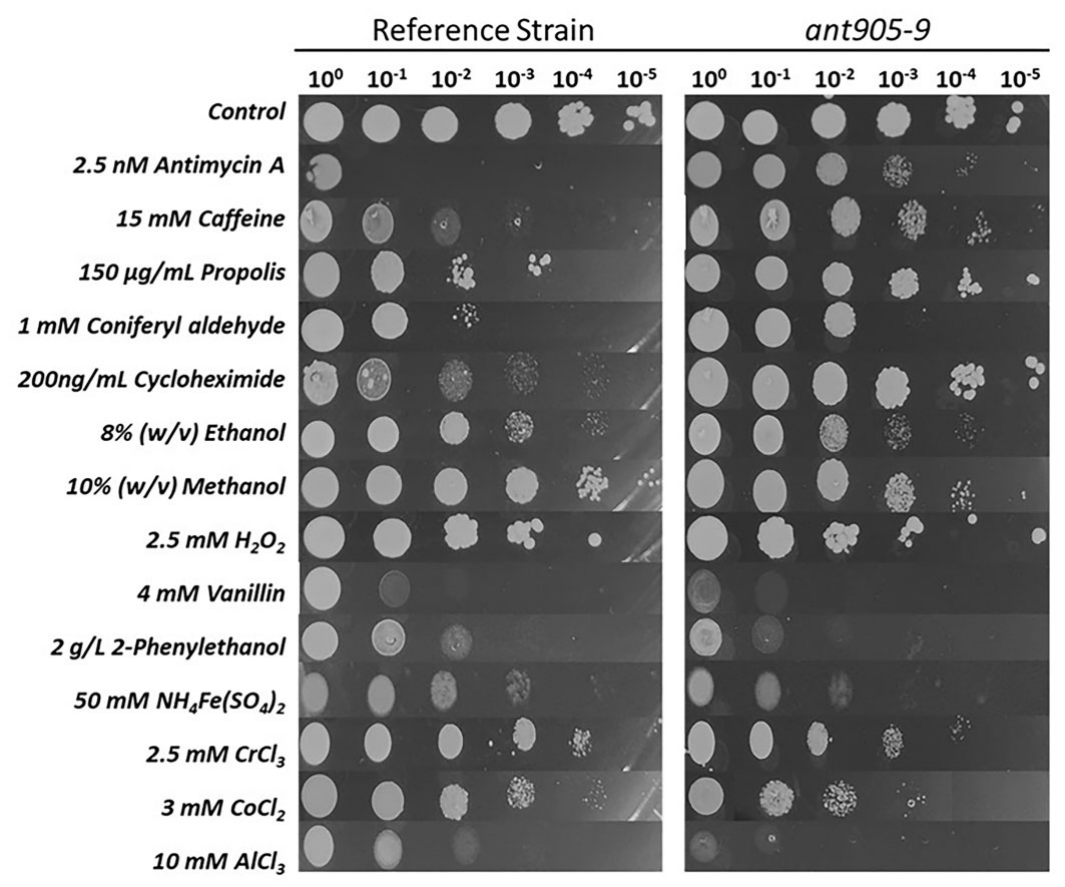
Spot assay results of the antimycin A-resistant *ant905-9* evolved strain and the reference strain, indicating their resistance and sensitivities against diverse stressors. Control indicates YMM plates without any stressor. Images were taken at the 72nd h of incubation at 30°C.

Improving a specific cellular property through evolutionary engineering can result in trade-offs in different traits (Çakar et al. 2012). To test for possible cross-resistance or sensitivities of the evolved strain *ant905-9* compared to the reference strain, spot assays were used on YMM agar plates supplemented with different stressors. The results revealed that the evolved strain was cross-resistant against caffeine, propolis, coniferyl aldehyde, and cycloheximide, all of which have been shown to be associated with PDR mechanisms in our previous study (Sürmeli et al. 2019, Özel et al. 2024). On the other hand, *ant905-9* showed remarkable sensitivity to aluminum and slight sensitivity to chromium, cobalt, and iron stresses (Fig. 2). Interestingly, the evolved strain, which was selected using ethanol as the sole carbon source, showed better growth than the reference strain under ethanol stress in respiratory growth conditions (data not shown), but not under respiro-fermentative conditions, as no cross-resistance was observed against ethanol stress (Fig. 2).

### Growth physiology and metabolite profiles of *ant905-9* and the reference strain

Batch cultivation and metabolite analysis were performed with the evolved strain *ant905-9* and the reference strain, under respiratory and fermentative growth conditions, using 0.67% (w/v) yeast nitrogen base without amino acids supplemented with 1% (w/v) ethanol or 2% (w/v) glucose as the sole carbon source, respectively. Since Antimycin A is known to be ineffective under fermentative conditions (Luzia et al. 2024), it was only applied under respiratory growth conditions.

**Figure 3.**
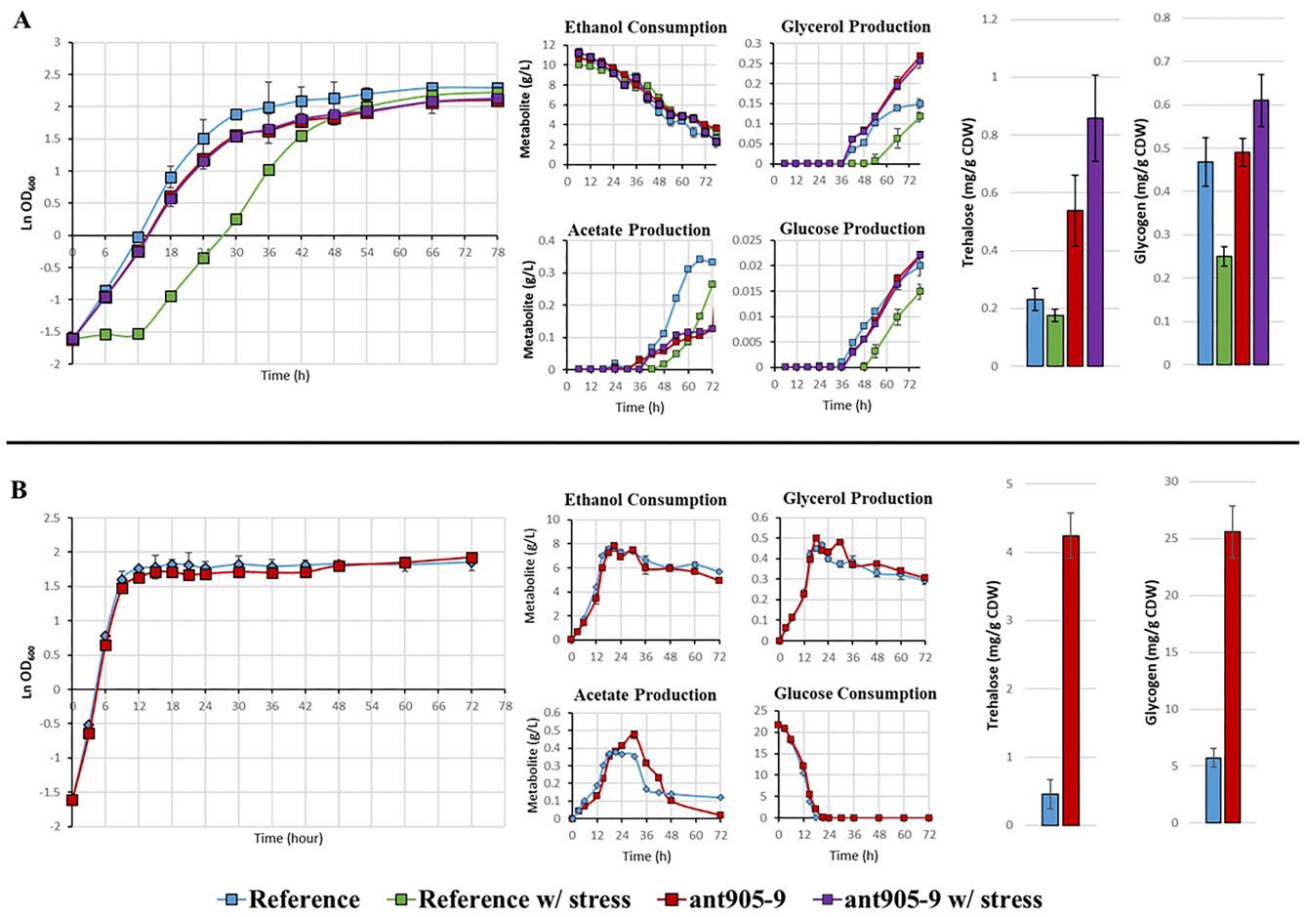
Growth and metabolite profiles of the evolved strain *ant905-9* and the reference strain under (A) respiratory and (B) fermentative growth conditions, and final trehalose and glycogen amounts (mg/g cell dry weight) of the cultures measured at the end of the cultivations.

Under respiratory growth conditions using ethanol as the sole carbon source, the reference strain could not grow above 1 nM of antimycin A concentration, while the evolved strain could cope with higher concentrations (Fig. S1). Thus, comparative growth physiological analysis of both strains was performed at 1 nM antimycin A-stress condition, and maximum specific growth rates of the evolved strain in the absence or presence of antimycin A-stress were found to be 0.25/h and 0.23/h, respectively, with no significant difference in growth. However, the maximum specific growth rate of the reference strain decreased under antimycin A-stress condition, from 0.34/h to 0.28/h, and the reference strain had a lag phase of about 12 h in the presence of antimycin A-stress, compared to control conditions. Glucose, acetate and glycerol production of the reference strain were also delayed under antimycin A stress, in line with the observed lag phase. Significant differences were observed in the metabolite profiles for glycerol and acetate, with the evolved strain producing more glycerol and less acetate than the reference stain, both in the presence and absence of antimycin A-stress (Fig. 3A). Under fermentative conditions, there was no significant difference between the maximum specific growth rates of both strains, which was about 0.85/h. Although similar profiles were observed between the evolved strain and the reference strain regarding ethanol and glycerol production and glucose consumption, the evolved strain *ant905-9* showed significant differences in acetate levels by producing more acetate than the reference strain and subsequently consuming all acetate produced during later stages of growth (Fig. 3B).

The accumulation of storage carbohydrates was also analyzed in the *ant905-9* strain in comparison with the reference strain. Both trehalose and glycogen amounts produced per gram of cell dry weight were significantly higher in the evolved strain under fermentative conditions, compared to the reference strain (Fig. 3B). On the other hand, under respiratory growth conditions, significantly lower amounts of storage carbohydrate production were observed in both reference and the evolved strain, compared to the fermentative conditions. The trehalose and glycogen amounts produced per gram of cell dry weight, however, were generally higher in the evolved strain, compared to the reference strain, both in the presence and absence of antimycin A-stress (Fig. 3A). In addition, the application of antimycin A-stress resulted in decreased storage carbohydrate production in the reference strain, but increased storage carbohydrate production in the evolved strain (Fig. 3B).

### Cell wall integrity analysis of the evolved strain *ant905-9*

**Figure 4.**
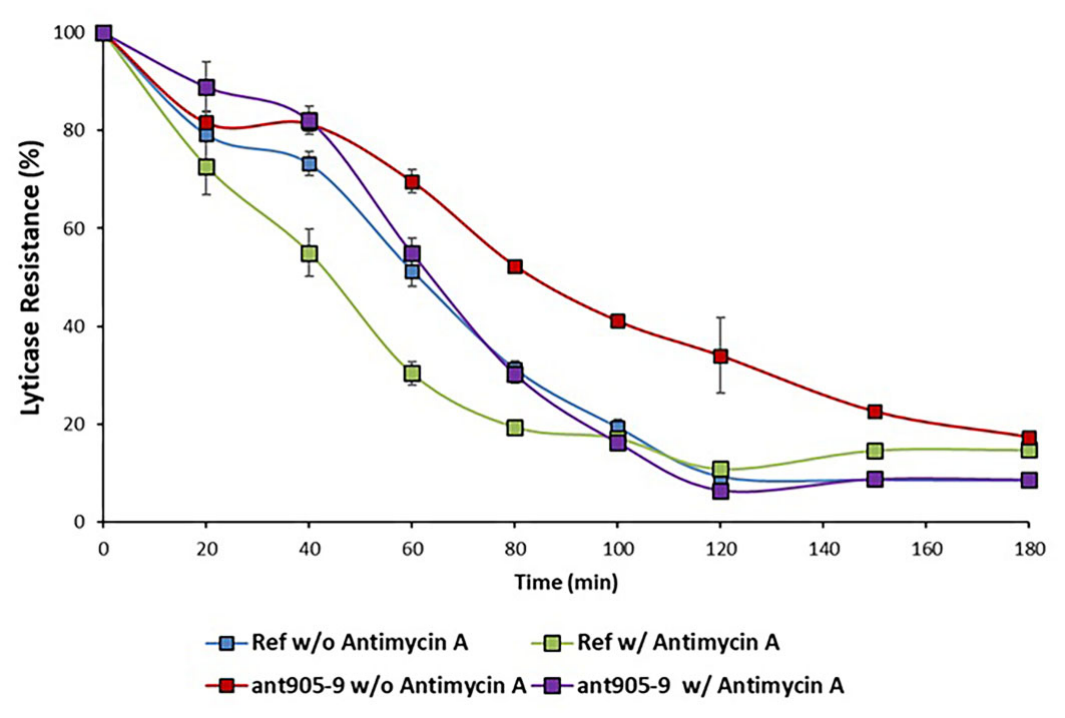
Lyticase sensitivity of *ant905-9* and the reference strain (Ref) in the presence and absence of 1 nM antimycin A-stress. Lyticase sensitivity was assessed as the % decrease in lyticase resistance, where the initial value was 100%.

To evaluate the cell wall integrity of *ant905-9* in comparison to the reference strain both in the presence and absence of 1 nM antimycin A-stress, a lyticase susceptibility test was applied using the cell wall (β1–3 glucan) perturbing enzyme lyticase, and by monitoring the decline in the absorbance to calculate % decrease in lyticase resistance. The results revealed that the evolved strain *ant905-9* had significantly higher lyticase resistance, indicating improved cell wall integrity than the reference strain, both in the presence and absence of antimycin A-stress. Both the evolved and the reference strains showed a decrease in the cell wall integrity, when exposed to antimycin A (Fig. 4).

### Whole genome resequencing of *ant905-9*

Comparative whole genome resequencing analysis generated 9.3 million reads and 153x mean depth of coverage for the *ant905-9* strain. Only two SNVs were identified in the *ant905-9* genome, compared to the reference strain. These transition mutations were found on *PDR1* and *PRP8* genes (Table 1).

**Table 1.** The SNVs found in the genome of the evolved strain *ant905-9*, compared to the reference strain.

Gene	Genetic change	Amino acid substitution	Description (Cherry et al. 2012)
*PDR1*	c.2195 T > G	M732R	Transcription factor that regulates pleiotropic drug response and is involved in the multidrug response (MDR) pathway
*PRP8*	c.6652 G > T	V2218L	Encodes the Prp8p protein, which is an evolutionarily conserved protein of the spliceosome.

The *PDR1* gene is found on the seventh chromosome of the yeast *S. cerevisiae* and codes for a transcription factor Pdr1p that regulates pleiotropic drug response and is involved in the multidrug response (MDR) pathway (Jungwirth and Kuchler 2006). The specified mutation Pdr1p.M732R was found on the regulatory domain of the Pdr1p protein and this missense mutation leads to an amino acid substitution from methionine to arginine. The other SNV was found in the *PRP8* gene which consists of 7242 bp and is located on the eighth chromosome of the *S. cerevisiae* genome. Prp8p protein is the largest and the most evolutionarily conserved protein of the spliceosome and occupies a central position in the catalytic core (Grainger and Beggs 2005). The Prp8p.V2218 L mutation was found on the MPN domain of Prp8p, which acts as a protein interaction domain (Boon et al. 2006).

### Reverse engineering of *PDR1* and *PRP8* gene mutations by CRISPR/Cas9 technology and mutant phenotyping

**Figure 5.**
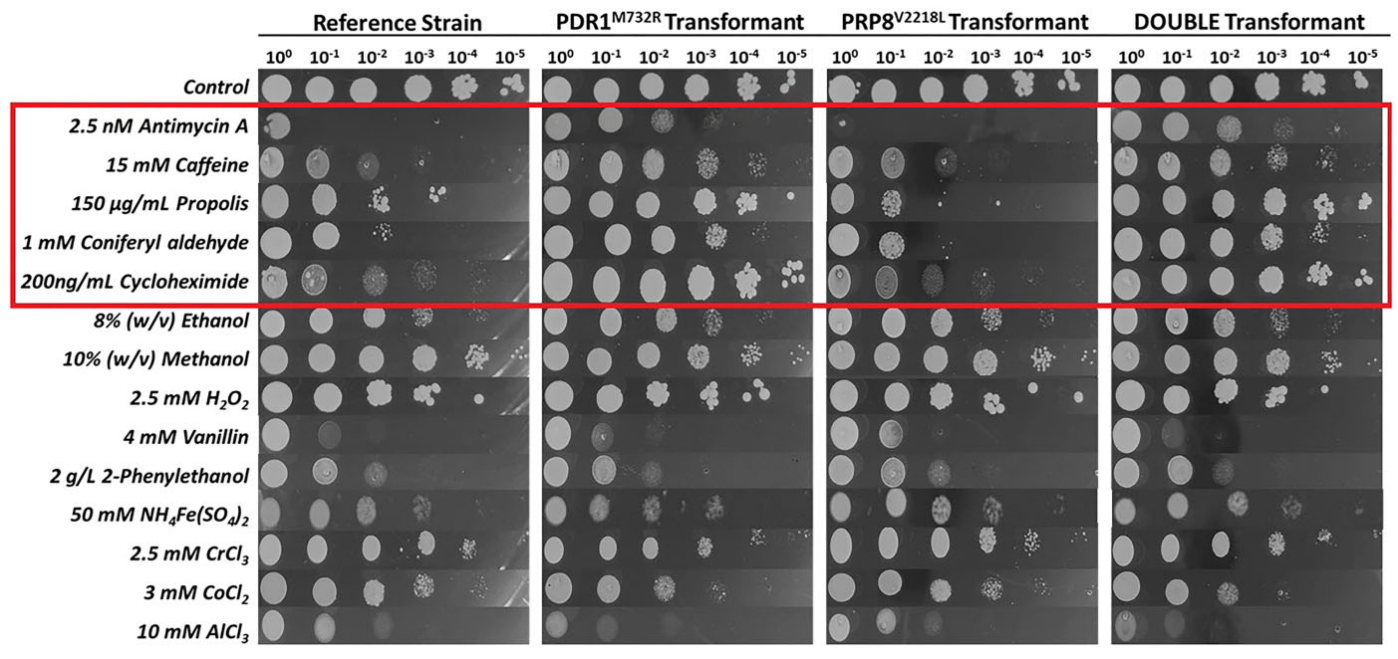
Spot assay results of the reference (unevolved) strain and its single transformants *PDR1*^M732R^, *PRP8*^V2218 L^, and the double transformant (*PDR1*^M732R^ and *PRP8*^V2218L^) obtained by reverse engineering, indicating their resistance and sensitivities against antimycin A and other stressors. Control indicates YMM plates without any stressors. Images were taken at the 72nd h of incubation at 30°C. The *PDR1*^M732R^ transformant strains were resistant to antimycin A, caffeine, propolis, coniferyl aldehyde, and cycloheximide, just as the evolved strain *ant905-9* (shown in a frame).

As only two genes, *PDR1* and *PRP8*, were found to have SNVs in the antimycin A-resistant, evolved strain *ant905-9*, reverse engineering by CRISPR/Cas9 technology was applied to transfer these variations into the reference strain and to test for antimycin A-resistance in the resulting single (*PDR1*^M732R^ or *PRP8*^V2218L^) and double (*PDR1*^M732R^ and *PRP8*^V2218L^) mutant strains. The spot assay results of the reference strain and the mutant strains in the presence of 2.5 nM antimycin A stress and various other stressors are shown in Fig. 5. The results revealed that the strain carrying the Pdr1p.M732R mutation was resistant to antimycin A-stress and showed cross-resistance against caffeine, propolis, coniferyl aldehyde, and cycloheximide, and sensitivity against aluminum, cobalt, and iron stresses, which are in line with the results of the evolved strain *ant905-9* (Figs 2 and 5). However, the transformant strain carrying the Prp8.V2218 L mutation was not antimycin A-resistant and its cross-resistance and sensitivity response against other stressors were similar to that of the reference strain, and not *ant905-9* (Figs 2 and 5). This implies that *PDR1*, but not *PRP8*, is involved in antimycin A-resistance.

### Comparative whole genome transcriptomic analysis of the Pdr1p.M732R mutant strain

**Figure 6.**
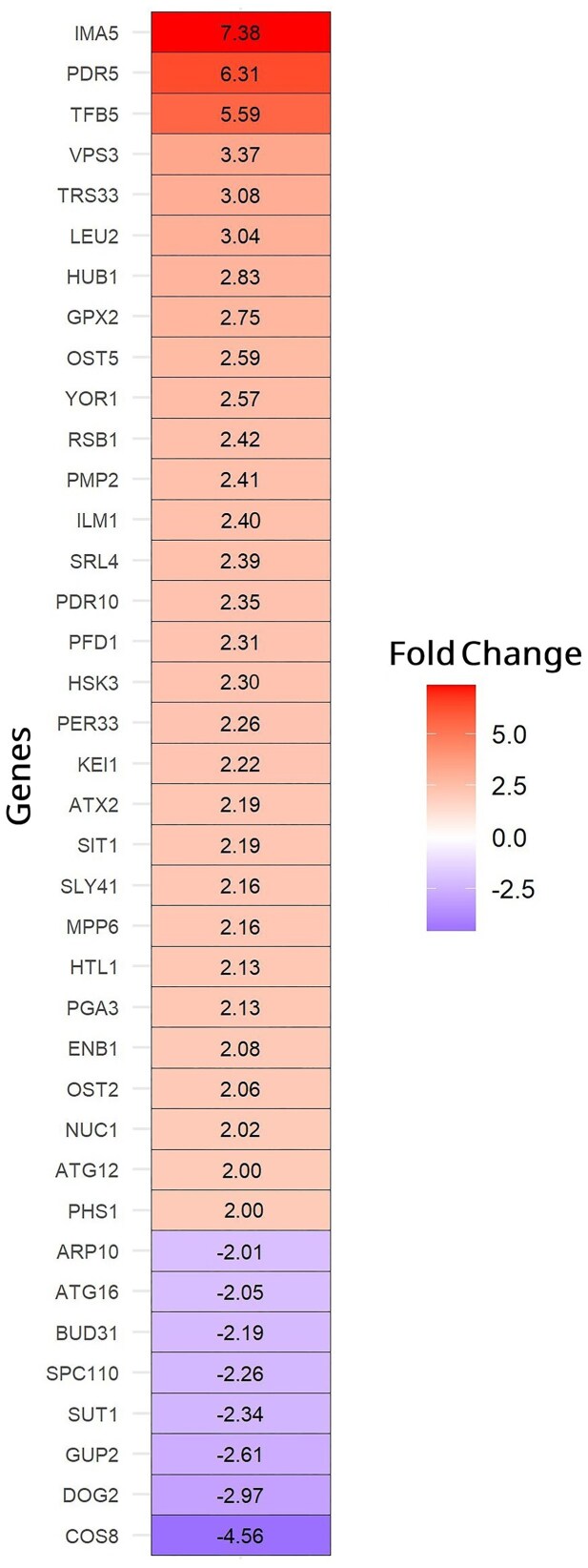
The differentially expressed genes in the reverse engineered, Pdr1p.M732R mutant strain (at least 2-fold change and adjusted *P*-values < .05), compared to the reference strain. Genes with increased transcript levels are shown in red, and the genes with decreased transcript levels are shown in violet.

To identify the transcriptomic changes associated with the *PDR1*^M732R^ point mutation, comparative transcriptomic analysis was performed using RNA sequencing technology with the reverse engineered, Pdr1p.M732R mutant and the reference strain. Because the evolutionary selection was carried out under respiratory growth conditions, we performed transcriptomic analyses under the same context to directly capture changes related to the *PDR1*^M732R^ mutation. We also chose to grow strains in the absence of antimycin A-stress, and sampled during mid-exponential growth phase, to observe the effects of the *PDR1* mutation without interference with antimycin A stress, and when the cells exhibit maximal metabolic activity. Based on *S. cerevisiae* CEN.PK113-7D (ASM26988v1) Ensembl Genome Assembly, from 5176 transcripts, we found that 30 genes were upregulated, and 8 genes were downregulated, when at least 2-fold change and adjusted *P*-values < .05 were used as the criteria for differential gene expression (Fig. 6).

The most upregulated genes in the Pdr1p.M732R mutant strain were *IMA5* and *PDR5* that were upregulated by 7.38-fold and 6.31-fold, respectively. *IMA5* encodes the enzyme alpha-glucosidase, which has specificity for isomaltose, maltose, and palatinose (Cherry et al. 2012). The most downregulated (4.56-fold) gene in the Pdr1p.M732R mutant strain was *COS8*, which is implicated in endosomal trafficking (MacDonald et al. 2015). The other two most downregulated genes, *GUP2* (2.61-fold) and *DOG2* (2.97-fold), are related to glycerol metabolism and osmotic stress, respectively (Holst et al. 2000, Tsujimoto et al. 2000).

According to KEGG pathway analysis, genes associated with ABC transporters were the most significantly enriched among the upregulated set (Table 2). The genes that belong to this category include *PDR5* (6.31-fold), *PDR10* (2.35-fold), and *YOR1* (2.57-fold), which were among the most upregulated genes in the Pdr1p.M732R mutant strain, compared to the reference strain.

**Table 2.** KEGG pathway analysis results of the upregulated genes in the reverse engineered, Pdr1p.M732R mutant strain, compared to the reference strain.

Term	Genes	Count^a^	%^b^	*P*-^c^value	Fold^d^ enrichment
*Upregulated genes*					
ABC transporters	*PDR5, PDR10, YOR1*	3	10	3.1 × 10^−3^	32.2

a Number of input genes involved in each term.

b Percentage of the involved genes among the total genes in the query.

c *P*-values were adjusted with Benjamini–Hochberg false discovery rate correction (Benjamini and Hochberg 1995).

d Ratio between frequencies of the query and the reference gene set.

The GO enrichment analysis revealed that several GO terms associated with biological function, cellular compartment and molecular function were significantly enriched (*P* < .05) among the at least 2-fold upregulated genes set of the Pdr1p.M732R mutant strain (Table 3).

**Table 3.** Significantly enriched (*P* < .05) GO terms (associated with biological function, cellular compartment, and molecular function) among the at least 2-fold upregulated genes set of the reverse engineered, Pdr1p.M732R mutant strain, compared to the reference strain.

Term	Genes	Count^a^	%^b^	*P*-^c^value	Fold^d^ enrichment
Biological function					
Transmembrane transport	*YOR1, ENB1, ATX2, SLY41, SIT1*	5	16.7	1.8 × 10⁻²	4.7
Autophagy	*ATG12, VPS3, TRS33*	3	10.0	4.7 × 10⁻²	8.3
Siderophore transmembrane transport	*ENB1, SIT1*	2	6.7	2.7 × 10⁻²	71.7
Xenobiotic detoxification by transmembrane export across the plasma membrane	*PDR10, PDR5*	2	6.7	4.0 × 10⁻²	47.8
Cellular compartment					
Membrane	*PDR10, PDR5, YOR1, ATG12, ENB1, ILM1, KEI1, ATX2, PER33, SLY41, PGA3, OST5, OST2, PHS1, RSB1, PMP2, SIT1*	17	56.7	1.0 × 10⁻³	2.1
Endoplasmic reticulum	*ILM1, ATX2, PER33, SLY41, TRS33, PGA3, OST5, OST2, PHS1, RSB1*	10	33.3	1.6 × 10⁻³	3.3
Plasma membrane	*PDR10, PDR5, YOR1, ENB1, PGA3, RSB1, PMP2, SIT1*	8	26.7	9.3 × 10⁻³	3.1
Cell periphery	*PDR10, PDR5, YOR1, ENB1, RSB1, PMP2*	6	20.0	7.6 × 10⁻³	4.6
Endoplasmic reticulum membrane	*ILM1, PER33, PGA3, OST5, OST2, PHS1*	6	20.0	3.1 × 10⁻²	3.2
Fungal-type vacuole	*PDR10, ENB1, PHS1, RSB1, SIT1*	5	16.7	3.7 × 10⁻²	3.8
Oligosaccharyltransferase complex	*OST5, OST2*	2	6.7	3.8 × 10⁻²	49.7
Molecular function					
ABC-type transporter activity	*PDR10, PDR5, YOR1*	3	10.0	4.3 × 10⁻³	29.3
ABC-type xenobiotic transporter activity	*PDR5, YOR1*	2	6.7	2.2 × 10⁻²	85.8
Siderophore-iron transmembrane transporter activity	*ENB1, SIT1*	2	6.7	2.7 × 10⁻²	71.5
Protein tag activity	*ATG12, HUB1*	2	6.7	4.4 × 10⁻²	42.9

a Number of input genes involved in each term.

b Percentage of the involved genes among the total genes in the query.

c *P*-values were adjusted with Benjamini–Hochberg false discovery rate correction (Benjamini and Hochberg 1995).

d Ratio between frequencies of the query and the reference gene set.

In line with the KEGG pathway analysis, “ABC-type transporter activity” was the most significantly enriched molecular function among the upregulated genes in the Pdr1p.M732R strain. Furthermore, “transmembrane transport” was the most significantly enriched biological process among the upregulated genes, and “Siderophore transmembrane transport,” “xenobiotic detoxification by transmembrane export across the plasma membrane,” and “autophagy” were the major biological functions in the upregulated genes of Pdr1p.M732R. The genes *VPS3* and *TRS33* belonging to the “Autophagy” category were highly overexpressed in the Pdr1p.M732R strain (3.37-fold and 3.08-fold, respectively). In accordance with the highly enriched transmembrane transport processes, 56.7% of the upregulated genes encode proteins related to the “membrane,” “endoplasmic reticulum,” and “vacuolar membranes” (Table 3). The GO enrichment analysis results also revealed that the genes encoding structural constituents of the cytoskeleton (*ARP10* and *SPC110*) were among the most enriched molecular function gene sets in the at least 2-fold downregulated genes of the Pdr1p.M732R mutant strain (data not shown). Together, these results strongly suggest that the *PDR1*^M732R^ point mutation promotes extensive transcriptional reprogramming toward enhanced membrane-associated transport and stress adaptation mechanisms, while downregulating cytoskeletal organization, potentially for membrane remodeling processes.

## Discussion

Investigation of the molecular mechanisms behind the resistance against a respirative inhibitor, antimycin A, can provide valuable information about antifungal drug resistance, an increasingly important issue in clinical research, and shed light on the regulatory changes in the central carbon metabolism of yeast, which are significant in biotechnological applications. In previous studies, antimycin A-resistant *S. cerevisiae* strains were isolated by chemical mutagenesis and/or selection on plates (Burger et al. 1976, Michaelis 1976, Lucchini et al. 1979). In this study, however, an antimycin A-resistant and genetically stable *S. cerevisiae* strain (*ant905-9*) was developed for the first time using evolutionary engineering, by systematic batch selection at gradually increased antimycin A concentrations, without prior chemical mutagenesis, and the molecular basis of antimycin A-resistance was elucidated at genomic and transcriptomic levels.

Antimycin A causes significant growth defects under respiratory growth conditions by binding to the Q_i_ site of the cytochrome bc_1_ complex by interrupting proton translocation (Huang et al. 2005). Our genetically stable, Antimycin A-resistant *ant905-9* strain could tolerate antimycin A levels higher than 25 nM in liquid YNBE, whereas the reference strain could not survive antimycin A levels above 1 nM. In addition, the evolved strain did not show a significant decrease in μ_max_ or an increase in the lag phase during growth in the presence of 1 nM antimycin A, where the reference strain was significantly inhibited (Fig. 3). The changes in the carbon and energy metabolism of the evolved strain were assessed through physiological and metabolic analyses on ethanol as the sole carbon source. The metabolite profiles of the evolved strain showed a considerable increase in glycerol but a decrease in acetate amounts under respiratory growth conditions (Fig. 3). This metabolic change might have resulted from the cells’ need to maintain the redox balance. The blockage of mitochondrial respiration through complex III, and growth on ethanol as the sole carbon, source could lead to the build-up of NADH and FADH_2_ in the cells (Scholz et al. 1969, Xiberras et al. 2019). To cope with this redox imbalance, metabolic flux might shift toward glycerol production rather than acetate to facilitate NADH oxidation under antimycin A stress (Cronwright et al. 2002). These changes demonstrate that the evolved strain adapts and optimizes its carbon metabolism and redox balance to cope with respirative inhibition and to sustain energy production.

The accumulation of storage carbohydrates was also analyzed in the *ant905-9* strain. Trehalose and glycogen are storage carbohydrates produced in response to environmental stresses in yeast (François and Parrou 2001). Trehalose and glycogen buildup were higher in the evolved strain in fermentative, nonstressful conditions, indicating a metabolism that is ready to withstand stressful environments. However, under respiratory growth conditions, both storage carbohydrates were produced in lower amounts, as glucose is a more efficient substrate than ethanol in trehalose and glycogen biosynthesis. Besides, under antimycin A stress, storage carbohydrate production decreased in the reference strain, while increased production was observed in the evolved strain. It is known that respiratory defects could harm glycogen production in *S. cerevisiae* (Wilson et al. 2002). However, the evolved strain stored more trehalose and glycogen in the presence of the respiratory inhibitor, antimycin A, suggesting molecular alterations for improved storage carbohydrate metabolism.

The cell wall integrity pathway is another pathway affected by environmental stress conditions (García et al. 2004). Although there are no reports in the literature about antimycin A directly affecting the cell wall integrity, our results indicate that the antimycin A-resistant evolved strain *ant905-9* has a stronger cell wall, compared to the reference strain, both in the presence and absence of antimycin A-stress. The increased cell wall integrity has been commonly observed in our previously obtained evolved strains that are resistant against caffeine (Sürmeli et al. 2019) and propolis stress (Demir-Yılmaz et al. 2025). Interestingly, *ant905-9* also gained cross-resistance against caffeine, propolis, coniferyl aldehyde, and cycloheximide (Fig. 2). These findings may suggest a common potential resistance mechanism between these stressors and antimycin A that may involve the cell wall integrity pathway. The lyticase susceptibility assay results of the present study also showed the negative effect of antimycin A stress on the cell wall integrity for both the reference and the evolved strain, although the latter was less affected (Fig. 4).

To elucidate the molecular mechanisms of antimycin A-resistance in the evolved strain, comparative genomic analysis was applied. Interestingly, comparative whole genome resequencing analysis revealed only two missense mutations in *ant905-9* genome that were found in *PDR1* and *PRP8* genes (Table 1). To verify the roles of the two SNVs (PDR1^M732R^ and PRP8^V2218L^) found in our evolved strain, these two mutations were introduced both individually and together into the background strain, using the CRISPR/Cas9 genome editing strategy. Cross-resistance analysis results revealed that the introduction of the *PDR1*^M732R^ mutation alone provided the reference strain with significant resistance to antimycin A and also cross-resistance against pleiotropic drugs, such as caffeine, propolis, coniferyl aldehyde, and cycloheximide, verifying the importance of the *PDR1* gene in PDR (Fig. 5). On the other hand, the transformant strain with the *PDR1* mutation showed remarkable sensitivity to aluminum and slight sensitivity to chromium, cobalt, and iron stress. It is already known that the PDR genes, especially *PDR1* and *PDR3*, can cope with metal toxicity caused by iron, cobalt, copper, cadmium, and arsenic in relation to Yap family transcription factors (Tuttle et al. 2003, Buechel and Pinkett 2020). However, the aluminum sensitivity observed in the Pdr1p.M732R strain in this study is yet to be investigated further, to unveil the potential relationship between aluminum resistance and the *PDR1* gene. Despite mitochondrial dysfunctioning, however, no sensitivity against oxidative stress (H_2_O_2_) was observed in the evolved strain *ant905-9* (Fig. 2).

Pdr1p regulates the ATP-binding cassette (ABC) transporter genes *PDR5, PDR10, SNQ2*, and *YOR1*, whose products promote drug efflux from the cell (Carvajal et al. 1997, Wolfger et al. 1997). It is known that the *PDR1* deletion typically results in increased sensitivity to a wide range of drugs, due to the loss of transcriptional activation of PDR genes (Fardeau et al. 2007). On the other hand, the multidrug ABC transporters display a high degree of overlapping substrate specificities. Sensitivity to some drugs was shown to be higher in pdr1Δ + pdr3Δ double deletion mutant of *S. cerevisiae* than the triple deletion mutant (pdr5Δ + snq2Δ + yor1Δ), suggesting the existence of additional, but unidentified, multidrug resistance mechanisms regulated by *PDR1/PDR3* (Rogers et al. 2001). It has also been recently reported that the point mutation *PDR1*^F749S^ was sufficient to provide strong protection of *S. cerevisiae* against fluconazole toxicity, whereas complete deletion of *PDR1* had no effect, underscoring that specific *PDR1* mutations can confer resistance through mechanisms distinct from simple loss-of-function, which is yet to be investigated in detail (Sánchez‐Adriá et al. 2025). Conversely, it was shown that the overexpression of the *PDR1* gene increased drug resistance by activating efflux pumps and other general protective mechanisms, but it also decreased cell growth (Yoshikawa et al. 2011). Unlike the results obtained in that *PDR1* overexpression study, the Pdr1p.M732R mutant strain in our study did not have a reduced growth rate (data not shown), despite an increase in its drug resistance. This may suggest that the mutations within the specific regions of the *PDR1* gene may show more controlled and targeted effects on the metabolism. Pdr1p has three functional domains; an N-terminal DNA-binding domain, a C-terminal activation domain, and a long internal region containing multiple inhibitory domains between these two domains extending from residue 435–1063 that shows an inhibitory effect on Pdr1p activation (Balzi et al. 1987, Kolaczkowska et al. 2002). Our evolved strain has a missense mutation (PDR1^M732R^) on the inhibitory domain, which could potentially decrease the inhibitory effect and cause elevated MDR.

**Figure 7.**
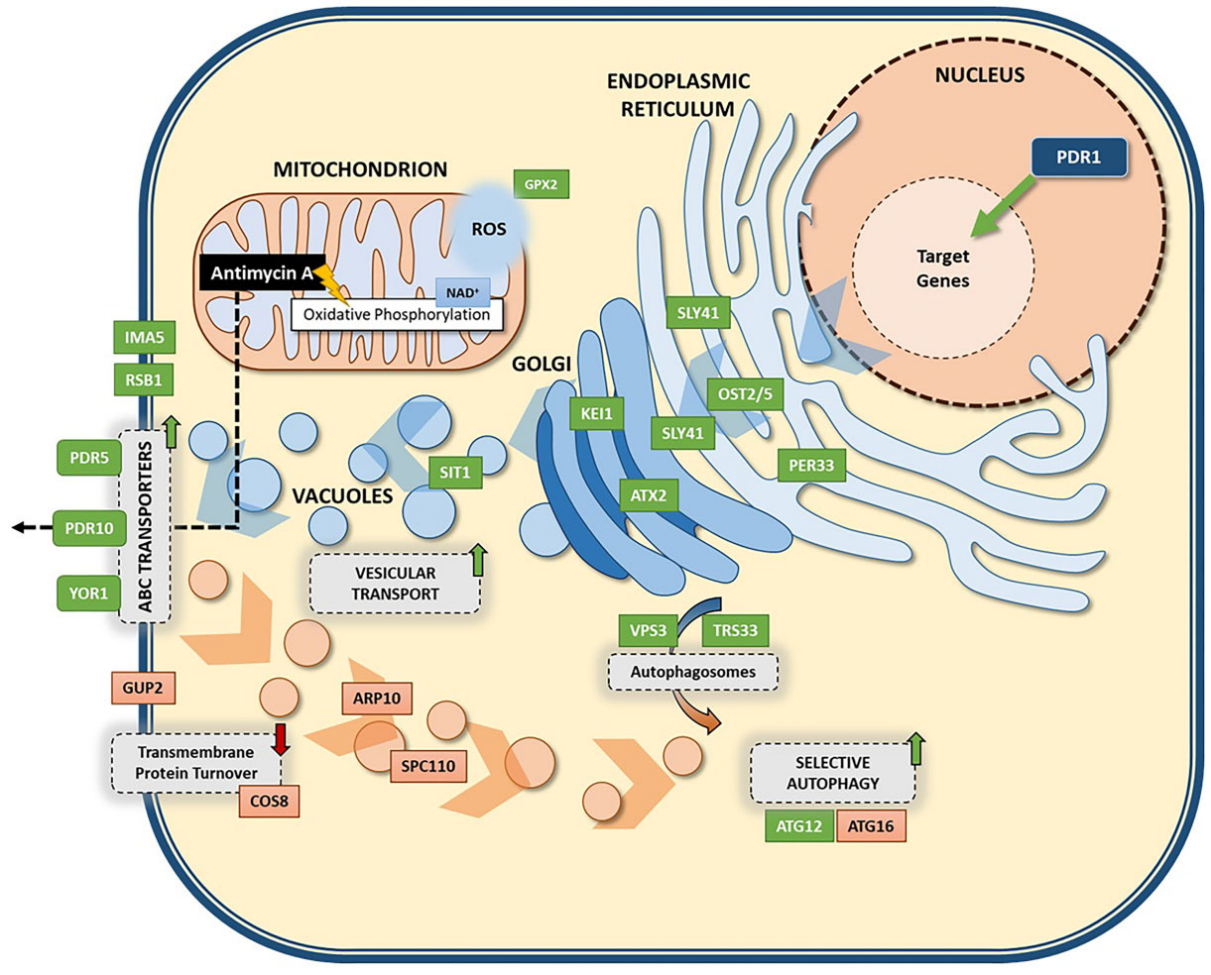
Major cellular processes that are differentially regulated in the reverse engineered, Pdr1p.M732R mutant strain under nonstress conditions, based on the transcriptomic analysis results. Green and orange boxes indicate up- and downregulated genes, respectively.

To identify the transcriptomic changes associated with the *PDR1*^M732R^ point mutation, comparative transcriptomic analysis was performed with the Pdr1p.M732R mutant and the reference strain, using RNA sequencing technology, in the absence of antimycin A-stress. The key transcriptomic changes are summarized in Fig. 7. The majority of the strongly upregulated genes in the Pdr1p.M732R mutant strain belonged to the ABC multidrug transporters: *PDR5* (6.31-fold), *PDR10* (2.35-fold), and *YOR1* (2.57-fold). According to our KEGG analysis results, those three genes (*PDR5, PDR10*, and *YOR1*) that are regulated by *PDR1* also belonged to the most enriched “ABC transporters” pathway in the upregulated gene sets of the Pdr1p.M732R mutant strain (Table 2), indicating an overall activation of the PDR pathway that may be associated with the antimycin A-resistance phenotype. In line with the ABC transporter activity, the GO analysis of the upregulated gene sets revealed that “transmembrane transport,” “siderophore transmembrane transport,” and “xenobiotic detoxification” were among the most significantly enriched biological processes. Furthermore, among the differentially expressed genes, *IMA5* exhibited the highest level of upregulation (7.38-fold). While *IMA5* is not directly associated with the PDR network, it has been recently reported that it is transcriptionally regulated by Pdr1p (Buechel and Pinkett 2024). *IMA5* is also associated with vacuolar morphology (Teste et al. 2010). The strong induction of *IMA5* in our Pdr1p.M732R mutant strain suggests a potential indirect role via the endomembrane system and vesicular trafficking in the PDR-related adaptive responses, which is yet to be investigated further.

Interestingly, genes involved in vesicular trafficking and endoplasmic reticulum organization, such as *VPS3, TRS33, OST2/5, PER33*, and *ILM1*, were also upregulated in the Pdr1p.M732R mutant strain, reflecting an increased demand for membrane and protein quality control systems, possibly due to an increased need for biogenesis, folding, and trafficking of ABC transporters during PDR activation (Raymond et al. 1990, Chavan et al. 2005, Lipatova et al. 2016). In parallel with the endomembrane activity, there were also highly upregulated genes in the Pdr1p.M732R strain that are associated with oxidative stress and autophagy, like *GPX2* (Inoue et al. 1999), *ATG12* (Mao and Klionsky 2011), vacuolar acidification (*VPS3*; Raymond et al. 1990), and preautophagosomal structure formation (*TRS33*; Lipatova et al. 2016). It is interesting to note that autophagy-related processes were also enriched selectively by the upregulation of *ATG12, VPS3*, and *TRS33*, but the downregulation of *ATG16*, possibly to mediate vesicle turnover and organelle-specific degradation.

Although most of the differentially regulated genes in the Pdr1p.M732R mutant strain were upregulated, structural constituents of cytoskeletal genes (*ARP10* and *SPC110*) were among the most enriched molecular function gene sets in its at least 2-fold downregulated genes, according to the GO enrichment analysis results (data not shown). The suppression of cytoskeletal elements, including *SPC110* and *ARP10*, might promote alterations in the intracellular architecture and membrane composition (Lyon et al. 2016, Clark and Rose 2006). In addition, the most downregulated (4.56-fold) gene in the Pdr1p.M732R mutant strain, *COS8*, encodes an endosomal protein involved in the plasma membrane protein turnover. Since COS proteins are known to accelerate the downregulation of a broad range of cell-surface proteins (MacDonald et al. 2015), the reduced expression of *COS8* in the Pdr1p.M732R mutant strain may contribute to the stabilization of plasma membrane transporters. Interestingly, the other highly downregulated gene in the Pdr1p.M732R mutant strain, *GUP2*, is responsible for plasma membrane organization and lipid homeostasis (Lucas et al. 2016). Thus, its downregulation might indicate a shift of the cellular activities away from membrane remodeling processes and toward maintenance of the ABC transporter activity at the cell surface.

For the first time in this study, a genetically stable, antimycin A-resistant *S. cerevisiae* strain was successfully developed using an evolutionary engineering strategy under respiratory growth conditions, and without prior chemical mutagenesis. The evolved strain sustained its growth even under mitochondrial dysfunction, and increased its glycerol production, indicating a potential for biotechnological applications. Comparative genomic analysis revealed a missense mutation in the *PDR1* gene and reverse engineering using CRISPR/Cas9 technology verified the role of *PDR1* in resistance to antimycin A and similar pleiotropic drugs, and aluminum sensitivity which is important for antifungal drug resistance. Transcriptomic profiling of the Pdr1p.M732R mutant strain revealed major alterations in ABC transporters, transmembrane transport, vesicular trafficking, and autophagy. Similar genomic and transcriptomic changes, including different SNVs in *PDR1, PDR5*, and *PDR10* genes and different upregulation profiles of ABC transporter genes, were also observed in our previously obtained, evolved *S. cerevisiae* strains that are resistant to caffeine (Sürmeli et al. 2019), coniferyl aldehyde (Hacısalihoğlu et al. 2019), and propolis (Demir-Yılmaz et al. 2025), but with different cross-resistance patterns. Considering that the Pdr1p.M732R mutant strain also showed cross-resistance against caffeine, coniferyl aldehyde and propolis stress, *PDR1* may be a common component in resistance against these stressors in *S. cerevisiae*. Taken together, our findings and the previous reports on *PDR1* (Rogers et al. 2001, Sánchez‐Adriá et al. 2025) highlight the complexity of *PDR1*-mediated resistance and suggest that the mechanism underlying the *PDR1*^M732R^ mutation likely involves multiple, overlapping pathways that remain to be fully elucidated. Based on those reports in the literature and our findings on the diverse stress-resistant evolved strains regarding the alterations in the *PDR1* gene and its downstream target genes, detailed investigation of our evolved strain collection is planned as a future study, by including targeted deletion studies of individual *PDR1*-related genes and their combinations, as well as the physiological, bioinformatic, and proteomic analyses of the resulting mutants. Such a comprehensive study would shed light on the specific contributions of individual transporters and regulatory networks to the observed resistance phenotypes.

To conclude, evolutionary engineering is a powerful strategy to develop stress-resistant and genetically stable *S. cerevisiae* strains, and reverse engineering strategies including genome editing by CRISPR/Cas9 strategy can help identify the complex genetic basis of desirable phenotypes such as stress resistance. The key role of *PDR1* and PDR in resistance against antimycin A was identified in this study, and is yet to be investigated further for antifungal drug applications to combat drug resistance issues.

## Supplementary Material

foaf062_Supplemental_Files

## Data Availability

Raw genomic data are available in the NCBI Sequence Read Archive (SRA) under BioProject PRJNA1166665. Transcriptome data are available NCBI Sequence Read Archive (SRA) database under the accession number SRP535472.
