# Supplementary material for: Evolutionary engineering and molecular characterization of an antimycin A-resistant Saccharomyces cerevisiae strain: the key role of pleiotropic drug resistance (PDR1)
Source: FEMS Yeast Res. 2025 Oct 17;25:foaf062. doi: 10.1093/femsyr/foaf062 (PMC12570882; doi:10.1093/femsyr/foaf062)
Supplement: foaf062_Supplemental_Files [file foaf062_supplemental_files.zip › Supplementary Figures.docx]

**SUPPLEMENTARY FILE**


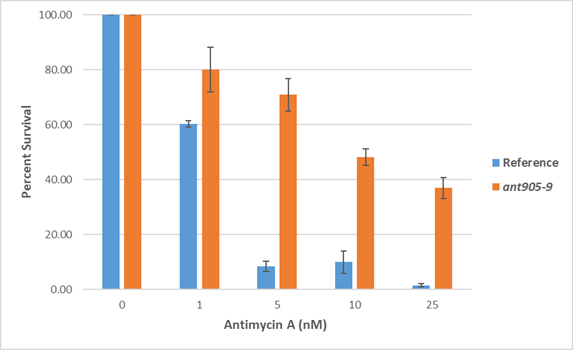


**Supplementary fig. 1:** Survival rates of the evolved strain *ant905-9* and the reference strain grown in YNBE medium supplemented with different concentrations of antimycin A.


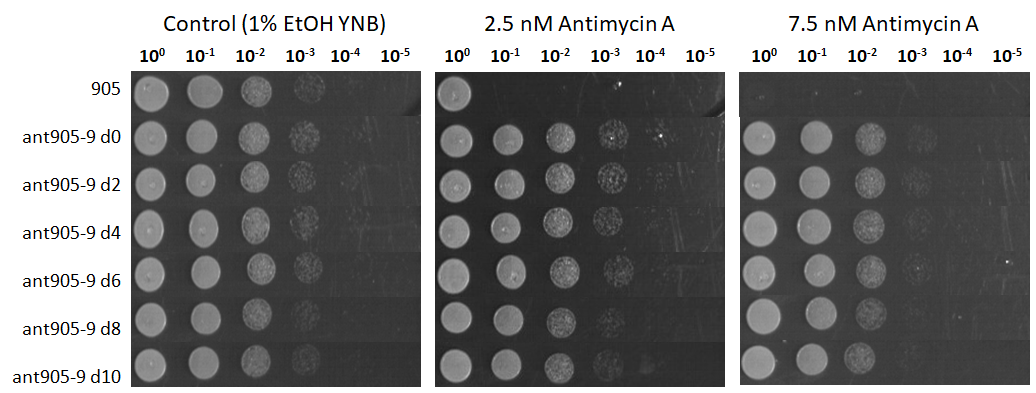


**Supplementary fig. 2: Genetic stability analysis of the evolved strain *ant905-9*.** The spot assay was applied to samples taken from the *ant905-9* culture cultivated in non-selective YMM for 10 days in YNBE agar plates supplemented with 2.5 and 7.5 nM of antimycin A. Photos were taken at 3^rd^ day of incubation.
